# Draft Genome Assembly of Rhodobacter sphaeroides 2.4.1 Substrain H2 from Nanopore Data

**DOI:** 10.1128/MRA.00414-20

**Published:** 2020-07-16

**Authors:** Robert Maximilian Leidenfrost, Nadine Wappler, Röbbe Wünschiers

**Affiliations:** a Department of Biotechnology and Chemistry, Mittweida University of Applied Sciences, Mittweida, Germany; University of Southern California

## Abstract

Rhodobacter sphaeroides is a purple bacterium with complex genomic architecture. Here, a draft genome is reported for R. sphaeroides strain 2.4.1 substrain H2, which was generated exclusively from Nanopore sequencing data.

## ANNOUNCEMENT

**FIG 1:**
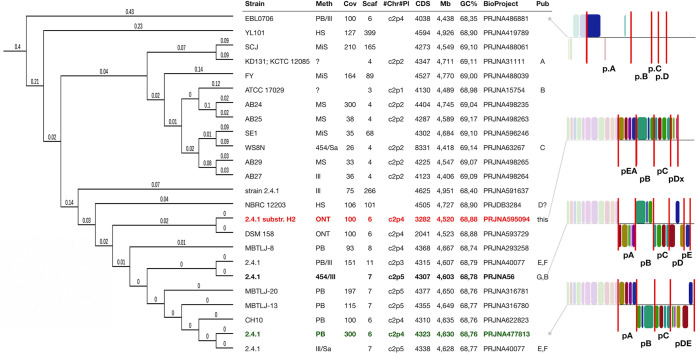
Compilation of available R. sphaeroides genome data. The BLAST tree display was created with NCBI Tree Viewer v1.17.6. It shows the autogenerated NCBI Genome Tree report (Tree 509) from genetic distances calculated from the aligned genome sequences using the Jukes-Cantor (16) substitution model. The tree was built from this distance matrix using FastME (17). BioProject number PRJNA376580 was used as an outgroup. The branch lengths are not to scale, and the numbers present percent genetic variation. In the table, substrain H2 and the former (PRJNA56) and current (PRJNA477813) reference strain 2.4.1 genomes are in bold. For the architecture diagrams, full-genome alignments for four strains were calculated with progressiveMauve v2.4.0 (18). Colored blocks represent homologous regions. Blocks below the center line indicate regions that align in the inverse orientation. Original plasmid labels were retained. While strain EBL0706 differed substantially, the rest were identical with respect to the chromosomes but differed in plasmid number and architecture. Meth, sequencing method; Cov, coverage; Scaf, scaffolds; #Chr#Pl, numbers of chromosomes and plasmids; CDS, coding sequences; Mb, genome size; GC%, GC content; Pub, related publication (A [19], B [20], C [21], D [22], E [23], F [24], G [3]); PB, PacBio single-molecule sequencing; Ill, Illumina; HS, Illumina HiSeq; MS, Illumina MiSeq; MiS, Illumina MiniSeq; 454, 454 GS FLX Titanium; ONT, ONT MinION; Sa, Sanger sequencing.

Rhodobacter sphaeroides 2.4.1 belongs to the phylogenetically distinct α-3 group of alphaproteobacteria and is capable of facultative photosynthesis. It has been well studied as a photosynthetic system and is considered the R. sphaeroides type strain. It was originally described by van Niel in 1944 (1), a near-complete genome was published by Mackenzie et al. in 2001 (2), and the sequence was revised by Kontur et al. in 2012 (3) (see BioProject accession number PRJNA56 in Fig. 1). Here, we report the Nanopore sequencing based *de novo* assembly for strain R. sphaeroides 2.4.1 substrain H2 (4), which is of particular interest and under current investigation within the context of photofermentative hydrogen production. It was acquired from TU Dresden and evolved serendipitously in the laboratory from the type strain (ATH 2.4.1; also named ATCC 17023, IAM 14237, and NCIB 8253), which was originally obtained as DSM 158 from the German Collection of Microorganisms and Cell Cultures (Braunschweig, Germany). For reference, we deposited the Nanopore raw read sequences of DSM 158 under SRA accession number SRX7341766.

Cultivation was performed in medium 112 (Van Niel’s yeast medium) at 33°C. Total DNA was extracted using the MasterPure total DNA purification kit (Epicentre).

Genomic DNA was sheared using g-TUBEs (Covaris) according to the protocol, purity was assessed using a NanoVue spectrophotometer (GE Healthcare), and the quantity was determined using a Qubit fluorometer with a high-sensitivity assay kit (Invitrogen). The library was prepared for 1D sequencing with an SQK-LSK108 kit (Oxford Nanopore Technologies [ONT]) and barcoded as part of a multiplexed sequencing run (EXP-NBD103; ONT). Sequencing was performed with an R9.4.1 flow cell (FLO-MIN106; ONT).

Base calling was performed with Guppy v3.0.3 (ONT), and results were further processed with Porechop v0.2.3 (https://github.com/rrwick/Porechop) (parameters: -b --barcode_threshold 85 --require_two_barcodes --discard_middle), i.e., demultiplexed and concurrently adapter trimmed. Read data quality was assessed with NanoPlot v1.27.0 (5). Assemblies were computed with Flye v2.5 (6) and consensus polished three times with Racon v1.4.3 (7); each contig was rotated and then signal-level polished twice with Nanopolish v0.11.2 (8). Starting points were adjusted to match those reported by Kontur et al. (3). Alignments were performed with minimap2 v2.11 (9). Polished assemblies were visually checked with Gepard v1.40 (10) and automatically annotated using PGAP upon submission to the NCBI database (11, 12). All processing was performed with default software parameters, unless otherwise specified.

Sequencing of 2.4.1 substrain H2 yielded 434 Mb of base-called and demultiplexed raw data (total number of reads, 35,752; mean length, 12,136 bp; *N*_50_, 23,716 bp), which were not further trimmed. The assembly is presented in six circular contigs, with an overall size of 4,519,621 bp. The contigs correspond to two chromosomes, 3.2 Mb and 0.9 Mb in size, and four plasmids, ∼124 kb, ∼114 kb, ∼105 kb, and ∼44 kb in size (Fig. 1). The sequence possesses an average coverage of 100×, an *N*_50_ value of 3,188,040 bp, and a mean GC content of 68.9%.

Notably, PGAP reports a large number of pseudogenes. These are reportedly caused by indels leading to frameshifts, which are known to be an error source in Nanopore sequencing-only assemblies (13) and must be taken into account for downstream purposes, such as annotation. While the plasmid architecture is substantially divergent from the sequence reported by Kontur et al. (3) (accession numbers ASM1290v2 and PRJNA56) (Fig. 1), such divergence may also be observed in the literature (14, 15) and for more recently published genome assemblies (accession numbers ASM332471v1/PRJNA477813 and ASM342926v1/PRJNA486881) (Fig. 1).

### Data availability.

The draft assembly of R. sphaeroides 2.4.1 substrain H2 has been deposited in NCBI GenBank under accession number ASM979766v1. The SRA deposit is available under accession number SRX7352322. The Nanopore raw read sequences of DSM 158 were deposited under SRA accession number SRX7341766.
